# Non-rheumatic calcific aortic valve disease in China: findings from the Global Burden of Disease study 2021

**DOI:** 10.3389/fcvm.2025.1664291

**Published:** 2026-01-16

**Authors:** Hongyi Tian, Zihua Liu, Junrui He, Kaiyue Sun, Yang You, Kai Liu

**Affiliations:** Department of Cardiovascular Surgery, Qilu Hospital of Shandong University, Shandong University, Jinan, China

**Keywords:** age–period–cohort model, China, disability-adjusted life years, epidemiology, Global Burden of Disease, non-rheumatic calcific aortic valve disease

## Abstract

**Background:**

Over the past 30 years, rapid population aging has made non-rheumatic calcific aortic valve disease (NR-CAVD) a growing public health concern in China. However, its long-term epidemiological trends and future disease burden remain unclear. This study aimed to comprehensively assess temporal trends in NR-CAVD in China from 1990 to 2021 and to predict its disease burden over the next 30 years from the Global Burden of Disease (GBD) 2021 data.

**Methods:**

Data on the incidence, prevalence, mortality, and disability-adjusted life years (DALYs) of NR-CAVD were retrieved from the GBD 2021 database, and estimated annual percentage changes (EAPCs) were calculated. Temporal patterns were evaluated using an age–period–cohort (APC) model, while Joinpoint regression was applied to identify key inflection points. Future trends were projected through a Bayesian APC (BAPC) model based on integrated nested Laplace approximation.

**Results:**

From 1990 to 2021, the age-standardized prevalence and incidence of NR-CAVD in China increased significantly (EAPC for prevalence: 2.33%, incidence: 2.03%), whereas mortality (EAPC = −1.34%) and DALYs (EAPC = −1.06%) showed a continuous decline. In 2021, adults aged ≥65 years accounted for more than two-thirds of total cases, with men showing higher age-standardized rates than women. Joinpoint analysis revealed an accelerated increase after 2005, and BAPC projections indicated that incidence will continue to rise through 2050, particularly among individuals aged 70–74 years.

**Conclusions:**

Although mortality and DALYs associated with NR-CAVD have gradually declined in China, the overall disease burden continues to increase due to population aging. Preventive strategies should prioritize adults over 50 years of age and integrate early screening alongside targeted interventions to reduce future cardiovascular risks.

## 1 Introduction

Non-rheumatic calcific aortic valve disease (NR-CAVD) is characterized by progressive calcification and thickening of the aortic valve leaflets, leading to stenosis and left ventricular outflow obstruction. With increasing life expectancy and a global shift from rheumatic to degenerative etiologies, NR-CAVD has emerged as a major contributor to cardiovascular morbidity worldwide (1, 2). Globally, calcific aortic valve disease has ranked among the fastest-growing causes of cardiovascular death and disability-adjusted life years (DALYs) in the past three decades (3, 4).

In China, the burden of valvular heart disease has expanded dramatically with population aging (5). National guideline data indicate a decline in rheumatic cases alongside a parallel increase in degenerative valve diseases among individuals aged 60 years and older (6, 7). According to the China Cardiovascular Health and Disease Report 2023, approximately 25 million people are currently living with valvular heart disease, with NR-CAVD constituting a growing share of this population. Despite its rising significance, epidemiological research on NR-CAVD in China remains fragmented and is often limited to specific provinces or single-year analyses.

Previous estimates from the Global Burden of Disease (GBD) 2019 study reported an increasing incidence of NR-CAVD in China; however, they did not fully characterize how age, period, and birth cohort effects jointly shape these observed trends (8, 9). Moreover, projections of the future disease burden, which are crucial for public health planning, remain scarce (10, 11). A comprehensive understanding of these patterns through modern epidemiological modeling may help clarify the drivers of NR-CAVD and inform age-targeted prevention strategies.

To address these gaps, we conducted a comprehensive analysis of NR-CAVD in China using data from the GBD 2021 study. Our objectives were threefold: (1) to quantify temporal trends in NR-CAVD prevalence, incidence, mortality, and DALYs from 1990 to 2021; (2) to disentangle the effects of age, period, and cohort using the APC model and identify turning points through Joinpoint regression; and (3) to forecast NR-CAVD burden in the next 30 years via Bayesian APC (BAPC) modeling and to identify high-risk subpopulations.

This work aims to provide robust evidence for national health policies and to guide future prevention, diagnosis, and management strategies for NR-CAVD in the context of China’s rapidly aging society.

## 2 Methods

### 2.1 Data sources and ethical considerations

NR-CAVD was defined according to the International Classification of Diseases, 10th Revision (ICD-10) codes. Data on NR-CAVD in China were obtained from the GBD 2021 database, curated by the Institute for Health Metrics and Evaluation (IHME). The regional search keywords in the database only cover disease control and prevention statistics from mainland China, excluding data from Hong Kong, Macao, and Taiwan regions of China. The GBD database provides comprehensive estimates of disease burden, including incidence, prevalence, mortality, and DALYs, stratified by age, sex, and year. All data are publicly available and de-identified; therefore, this study was exempt from ethical review and informed consent, in accordance with the Declaration of Helsinki (2013 revision).

To ensure comparability across time, all estimates were age-standardized to the GBD global reference population. Confidence intervals (CIs) were calculated at the 95% level to quantify estimation uncertainty. For contextual comparison, we also extracted global and East Asian regional data.

### 2.2 Epidemiological indicators

Four main indicators were analyzed: (1) prevalence: the number of existing NR-CAVD cases per 100,000 population; (2) incidence: the number of new NR-CAVD cases per 100,000 population per year; (3) mortality: the number of deaths attributed to NR-CAVD per 100,000 population (in the figures or charts, the term “deaths” from the GBD database is used to refer to “mortality”); and (4) DALYs: the sum of years of life lost (YLLs) and years lived with disability (YLDs) per 100,000 population.

To quantify long-term changes, we calculated the estimated annual percentage change (EAPC) for each indicator based on a log-linear regression model:

$$\text{EAPC}=100\times (e^{\beta }-1)$$

where β represents the slope of the natural logarithm of the age-standardized rate (ASR) over time. A positive EAPC indicates an increasing trend, while a negative EAPC indicates a decreasing trend. The trend was considered statistically significant if the 95% CI of EAPC did not include zero.

### 2.3 Age–period–cohort analysis

We employed the APC framework to disentangle the independent effects of age, period, and birth cohort on NR-CAVD incidence and prevalence from 1990 to 2021. The APC model estimates the following: (1) net drift—overall annual percentage change in the outcome across all age groups; (2) local drift—age-specific annual percentage change; (3) age effect—variation in disease risk across all age groups after controlling for period and cohort effects; (4) period effect—temporal influence affecting all age groups simultaneously (e.g., diagnostic improvements or policy changes); and (5) cohort effect—generational changes associated with birth year and lifetime exposure to risk factors.

Relative risk (RR) values were calculated for each dimension, using the 1992–1996 period and the 1972 birth cohort as reference categories. All APC analyses were conducted using the Web-based APC tool developed by the U.S. National Cancer Institute (12).

### 2.4 Joinpoint regression analysis

To identify inflection points and quantify temporal shifts in NR-CAVD burden, we applied Joinpoint regression (version 4.9.1.0, U.S. National Cancer Institute). This model fits a series of connected linear segments to log-transformed rates over time, allowing for the detection of statistically significant changes in trend direction (“joinpoints”) (13, 14).

For each segment, the annual percentage change and its 95% CI were estimated. The average annual percentage change (AAPC) was then computed as a summary measure for the entire study period. In this study, the Joinpoint analysis focused primarily on the 50–80-year-old population, as this age range was identified as high risk based on the APC analysis.

### 2.5 Bayesian age–period–cohort forecasting model

Future trends in the NR-CAVD burden (2022–2050) were projected using a BAPC model, implemented within the integrated nested Laplace approximation (INLA) framework (15, 16). The BAPC model extends traditional APC analysis by integrating Bayesian inference to estimate posterior distributions of rates and to smooth variability across age, period, and cohort dimensions. This approach provides more stable and interpretable long-term forecasts than classical regression methods, particularly when recent data are sparse or volatile.

We generated projections for both the total population and a stratified subgroup aged 50–80 years, which represents the demographic with the highest observed incidence and prevalence.

### 2.6 Statistical analysis

All rate-related data are presented as age-standardized rates (ASRs). All statistical analyses were conducted using R software (version 4.4.2) and Stata/MP 17.0. Descriptive statistics were used to summarize age-specific and sex-specific trends. Statistical significance was defines as P<0.05. For graphical representation, we used the ggplot2 package and the Joinpoint Regression Program for temporal trend analysis, while Bayesian modeling was conducted using the INLA package.

## 3 Results

### 3.1 Overall disease burden of NR-CAVD in China (1990–2021)

Between 1990 and 2021, the overall burden of non-rheumatic calcific aortic valve disease (NR-CAVD) in China increased substantially.

In 2021, the estimated number of NR-CAVD cases in China reached 685,550 (95% CI: 527,565–848,321), corresponding to an prevalence age-standardized rate of 32.68 per 100,000 (95% CI: 25.06–40.23).The EAPC of prevalence was 2.33% (95% CI: 2.22%–2.43%), which was comparable to the overall rate in East Asia (2.31%) but markedly higher than the global average (0.88%, Table 1).

**Table 1:** Prevalence of NR-CAVD in 2021 across different locations and SDI categories.

Location	Case prevalence	ASR prevalence	EAPC prevalence 1990–2021
China	685,550 (527,565–848,321)	32.68 (25.06–40.23)	2.33% (2.22%–2.43%)
Global	13,320,896 (11422,539–15249,410)	158.35 (135.92–181.00)	0.88% (0.78%–0.99%)
East Asia	719,161 (554,740–884,832)	33.15 (25.50–40.55)	2.31% (2.21%–2.41%)
Low SDI	85,816 (68,867–104,073)	17.64 (14.26–21.19)	0.75% (0.69%–0.8%)
Low-middle SDI	496,397 (404,011–589,829)	36.62 (29.46–43.57)	1.26% (1.22%–1.29%)
Middle SDI	1,417,738 (1,134,289–1,688,923)	54.49 (43.43–64.98)	1.75% (1.67%–1.82%)
High-middle SDI	3,440,879 (2,962,648–3,931,536)	172.89 (149.52–197.05)	1.38% (1.25%–1.5%)
High SDI	7,860,601 (6,819,445–8,892,888)	349.64 (303.58–395.77)	1.11% (1%–1.23%)

SDI, socio-demographic index. ASR: per 100,000.

Similarly, the ASR of incidence in 2021 was 2.52 per 100,000 (95% CI: 1.95–3.06), with an EAPC of 2.03% (95% CI: 1.96%–2.11%). In contrast, the ASR of mortality declined to 0.07 per 100,000 (95% CI: 0.06–0.10), corresponding to an EAPC of −1.34%, while DALYs showed a modest decrease (EAPC = −1.06%, 95% CI: −1.22% to −0.90%, Supplementary Tables S2, S3).

Overall, these data suggest that while improvements in clinical care and diagnostics may have contributed to reductions in mortality, the prevalence and incidence of NR-CAVD have continued to rise due to population aging and improved disease detection.

### 3.2 Age- and sex-specific distribution in 2021

**Figure 1:**
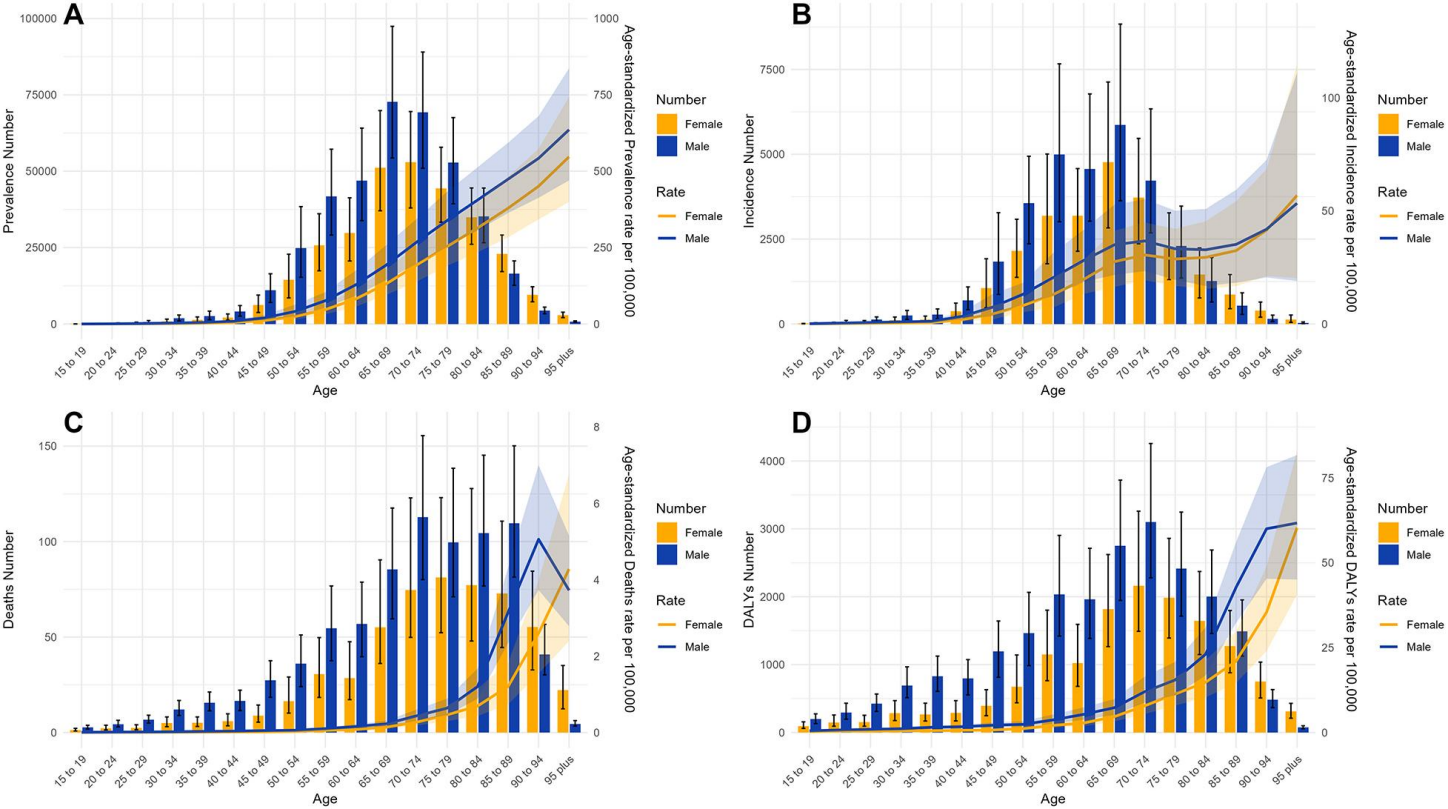
The ASR epidemiological parameters of NR-CAVD for age groups in China, compared by gender in 2021. Bars: number of cases in each age group. Error bars: 95% CI of the case number. Line: ASR parameters. Shaded area: 95% CI for the ASR. (**A**) prevalence, (**B**) incidence, (**C**) deaths, and (**D**) DALYs.

Stratified analyses by age and sex demonstrated a clear age-dependent increase in the burden of NR-CAVD (Table 2, Figure 1).

**Table 2:** Prevalence of NR-CAVD by age group in China in 2021.

Age	Case prevalence	ASR prevalence	EAPC prevalence 1990–2021
15–19	50 (26–81)	0.07 (0.04–0.11)	1.9% (1.8%–2%)
20–24	330 (171–536)	0.45 (0.23–0.73)	2.03% (1.93%–2.13%)
25–29	1,027 (533–1,680)	1.19 (0.62–1.94)	2.08% (1.96%–2.2%)
30–34	2,728 (1,399–4,480)	2.25 (1.16–3.70)	2.05% (1.91%–2.2%)
35–39	3,915 (2,002–6,432)	3.69 (1.89–6.07)	2.03% (1.9%–2.17%)
40–44	6,137 (3,814–9,255)	6.70 (4.17–10.11)	2% (1.9%–2.09%)
45–49	17,177 (10,908–26,051)	15.57 (9.89–23.61)	2% (1.92%–2.09%)
50–54	39,297 (23,971–61,412)	32.52 (19.83–50.81)	2.07% (1.96%–2.18%)
55–59	67,547 (46,869–93,176)	61.44 (42.63–84.75)	2.18% (2.05%–2.31%)
60–64	76,748 (55,245–104,178)	105.13 (75.67–142.70)	2.27% (2.13%–2.4%)
65–69	123,892 (91,774–168,075)	161.52 (119.65–219.12)	2.39% (2.26%–2.52%)
70–74	122,298 (90,070–158,420)	229.47 (169.00–297.24)	2.51% (2.39%–2.64%)
75–79	97,282 (72,549–124,880)	293.74 (219.06–377.07)	2.53% (2.41%–2.64%)
80–84	70,143 (52,956–89,047)	354.41 (267.57–449.92)	2.44% (2.35%–2.53%)
85–89	39,410 (29,722–49,798)	413.72 (312.02–522.78)	2.23% (2.17%–2.29%)
90–94	13,957 (10,621–17,598)	476.04 (362.26–600.21)	1.96% (1.93%–1.99%)
95 plus	3,604 (2,643–4,847)	563.94 (413.55–758.55)	1.74% (1.67%–1.81%)

ASR: per 100,000.

The highest number of prevalent cases was observed in the 65–69-year age group (123,892 case, Table 2), whereas the greatest increase in prevalence (EAPC = 2.53%) was observed in the 75–79-year age group. The corresponding peak in incidence (10,629 new cases) was also observed in the 65–69-year age group (Supplementary Table S4). In contrast, mortality shifted toward older age groups, reaching its maximum in the 70–74-year age group, with 187 deaths (95% CI: 142–255, Supplementary Table S5).

Across all age groups, men consistently exhibited higher ASRs for both incidence and mortality than women, although this disparity narrowed in the oldest age groups (Figure 1). DALYs rose sharply after age 60, peaking above 80 years, indicating that advanced age remains the dominant driver of the disease burden.

These findings identify individuals aged ≥65 years, particularly older men, as a key population for targeted prevention and screening strategies.

### 3.3 Age–period–cohort analysis

**Figure 2:**
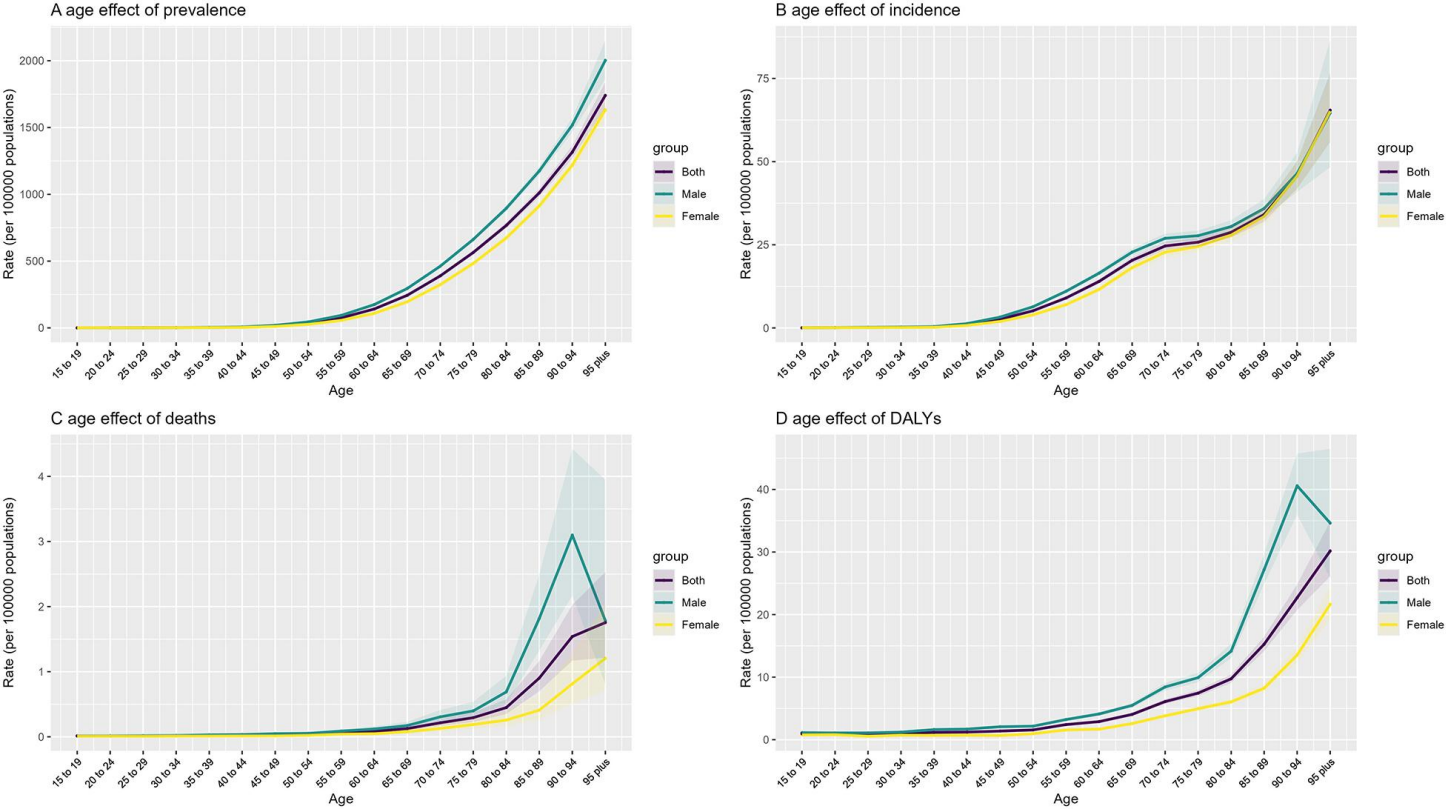
Age effect of the APC model, compared by gender in 2021. Obvious sex-stratified disparities are evident for both mortality rates and DALYs, with levels in men aged ≥65 years markedly higher than in women. Details on the remaining period and cohort effects are provided in the Supplementary Material. (**A**) prevalence, (**B**) incidence, (**C**) deaths, and (**D**) DALYs.

The age–period–cohort (APC) analysis revealed that all three dimensions—age, calendar period, and birth cohort—contributed to the observed trends in NR-CAVD (Figure 2).

Age effect: A sharp increase in the incidence and prevalence of NR-CAVD was observed among individuals aged 50 years and above, rising from 2.6 to 25.8 per 100,000 between ages 45 and 79 years. Mortality was consistently higher in men across age groups, with the largest sex disparity observed between 70 and 80 years. DALYs increased rapidly after 60 years of age, reflecting a cumulative burden of aging-related pathological processes.

Period effect: Using 1992–1996 as the reference, the RR of NR-CAVD incidence increased steadily after 1996, from 1.07 (95% CI: 1.04–1.10) in 1997–2001 to 1.59 (95% CI: 1.53–1.64) in 2017–2021. Meanwhile, the RR of mortality declined over the same period, reaching 0.68 (95% CI: 0.61–0.76).

Cohort effect: Individuals born after 1972 exhibited a progressive rise in NR-CAVD risk, with cohort-specific RR increasing from 1.10 (95% CI: 1.03–1.18) to 1.84 (95% CI: 1.13–3.00) in the 2006 birth cohort.

The APC model suggests that both population aging and generational exposure to cardiovascular risk factors have contributed to the upward trend of NR-CAVD in China.

### 3.4 Joinpoint analysis: identification of turning points

**Figure 3:**
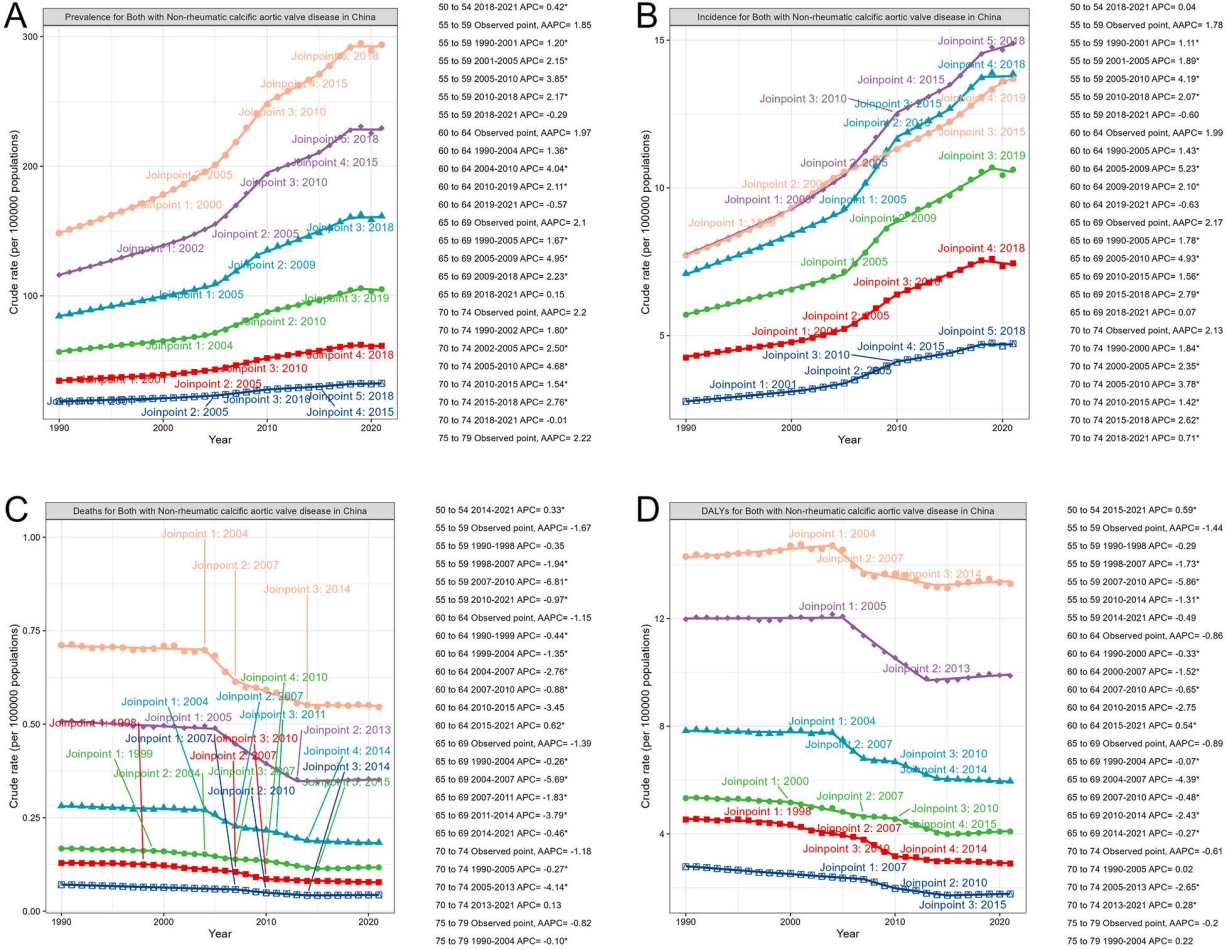
Turning point of epidemiological parameters predicted by the Joinpoint model of NR-CAVD in China, compared by age groups. The order of the lines from top to bottom represents descending age order: orange: 75–79 years; purple: 70–74 years; light blue: 65–69 years; green: 60–64 years; red: 55–59 years; dark blue: 50–54 years. (**A**) prevalence, (**B**) incidence, (**C**) deaths, and (**D**) DALYs. The determination of the number of change points adopts the Monte Carlo permutation test. We set the maximum potential number of change points to 5 and the minimum to 0. The permutation test starts with the number of change points k=0 and $k_{\max}=5$. If $k\neq k_{\max}$, then set k=k+1 and continue the test until the model corresponding to $k=k_{\max}$ is selected as the optimal model.

Joinpoint regression identified several significant inflection points in the 30-year trend of NR-CAVD burden (Figure 3).

Among individuals aged 50–80 years, both prevalence and incidence showed a marked acceleration beginning around 2005, with annual percentage changes exceeding 4.9% during 2005–2010 (prevalence: 4.95%, 95% CI: 4.72–5.12, P<0.05; incidence: 4.93%, 95% CI: 4.38–5.71, P<0.05).

In contrast, the DALY trend among individuals aged 70–74 years exhibited the most notable improvement between 2005 and 2013 (APC = −2.65%, 95% CI: −2.75 to −2.54, P<0.05), likely reflecting advancements in valve intervention and management. However, a modest rebound in DALYs was observed from 2013 to 2021 (APC = 0.28%, 95% CI: 0.14–0.4, P<0.05).

The Joinpoint results highlight 2005 as a key epidemiological turning point, coinciding with the expansion of echocardiographic screening and improved access to healthcare services for older adults in China.

### 3.5 Bayesian projections for the next 30 years (2022–2050)

**Figure 4:**
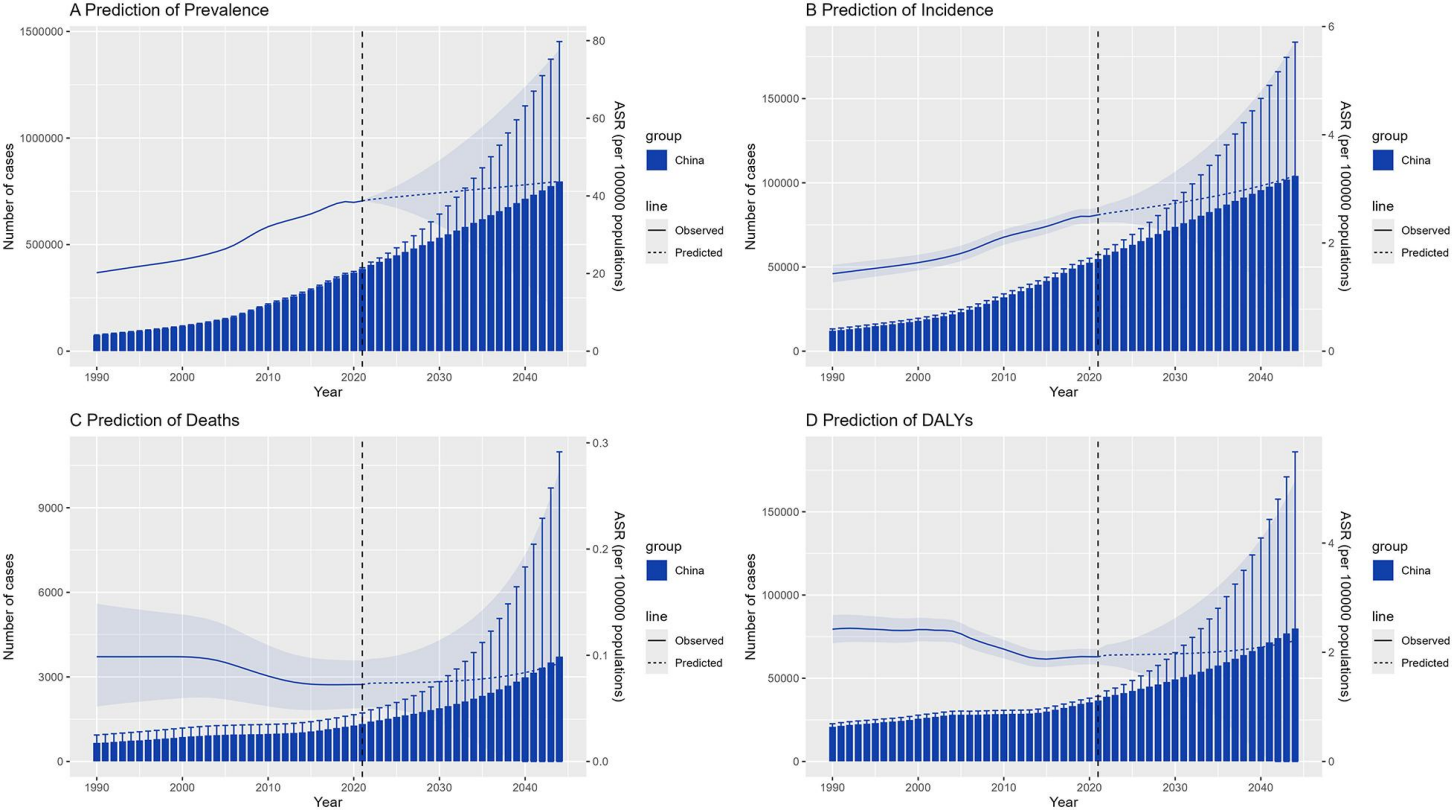
The trend of epidemiological parameters predicted by BAPC model of NR-CAVD for 30 years in the future. The bars represent the projected number of cases, the lines indicate the age-standardized rates, and the error bars and shaded areas denote the 95% CI. (**A**) prevalence, (**B**) incidence, (**C**) deaths, and (**D**) DALYs.

Using the Bayesian age–period–cohort (BAPC) model, we forecasted NR-CAVD trends through 2050 (Figure 4).

**Figure 5:**
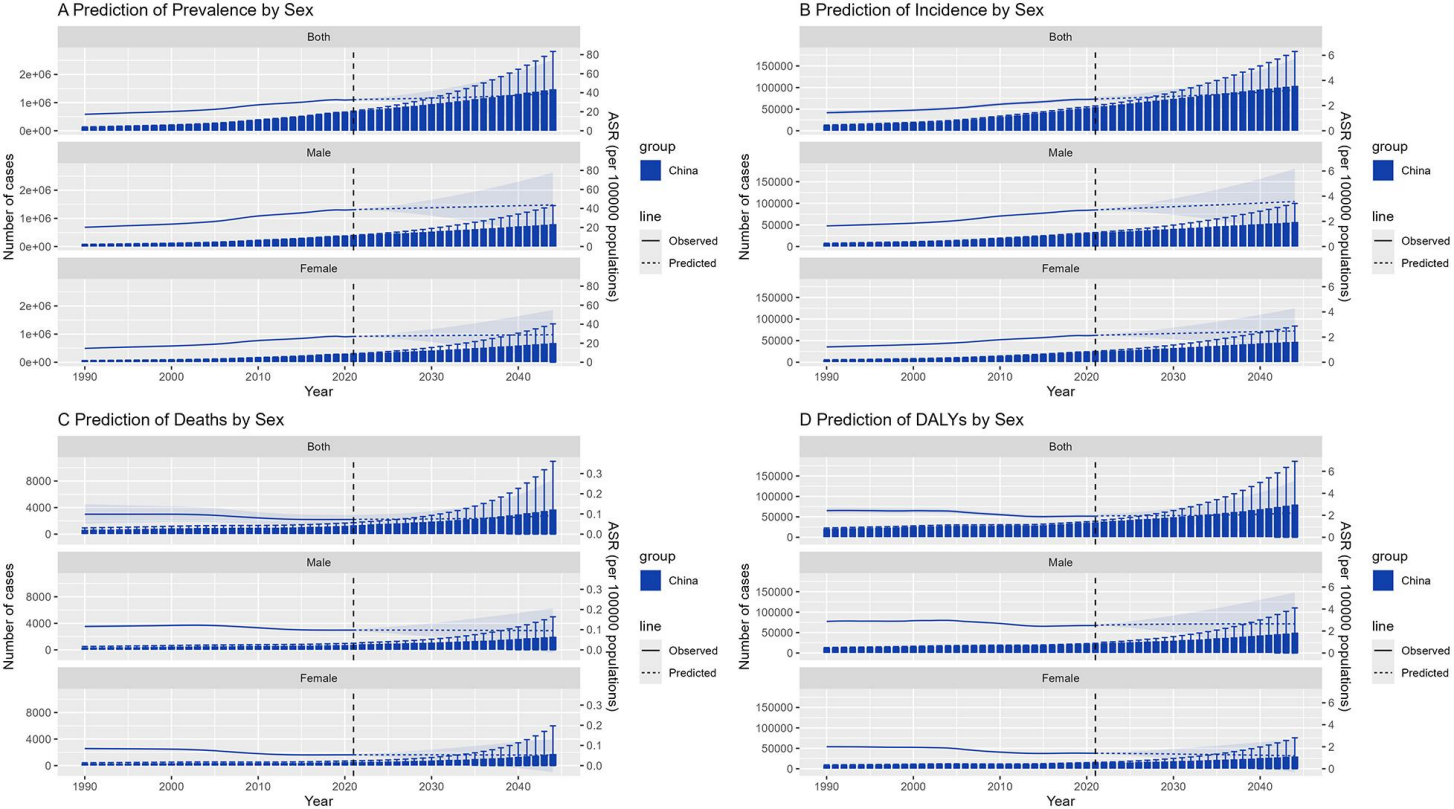
Prediction of NR-CAVD by the BAPC for sex groups in China, including the 50–80-year age group. In the figure, each epidemiological indicator is stratified by sex. The bars represent the projected number of cases, the lines indicate the age-standardized rates, and the error bars and shaded areas denote the 95% CI. (**A**) prevalence, (**B**) incidence, (**C**) deaths, and (**D**) DALYs.

The projections suggest that incidence and prevalence will continue to rise gradually, whereas mortality and DALYs are projected to remain relatively stable. By 2050, the total number of individuals living with NR-CAVD in China is projected to exceed 11 million, with approximately 6 million men and 5 million women affected (Figure 5).

**Figure 6:**
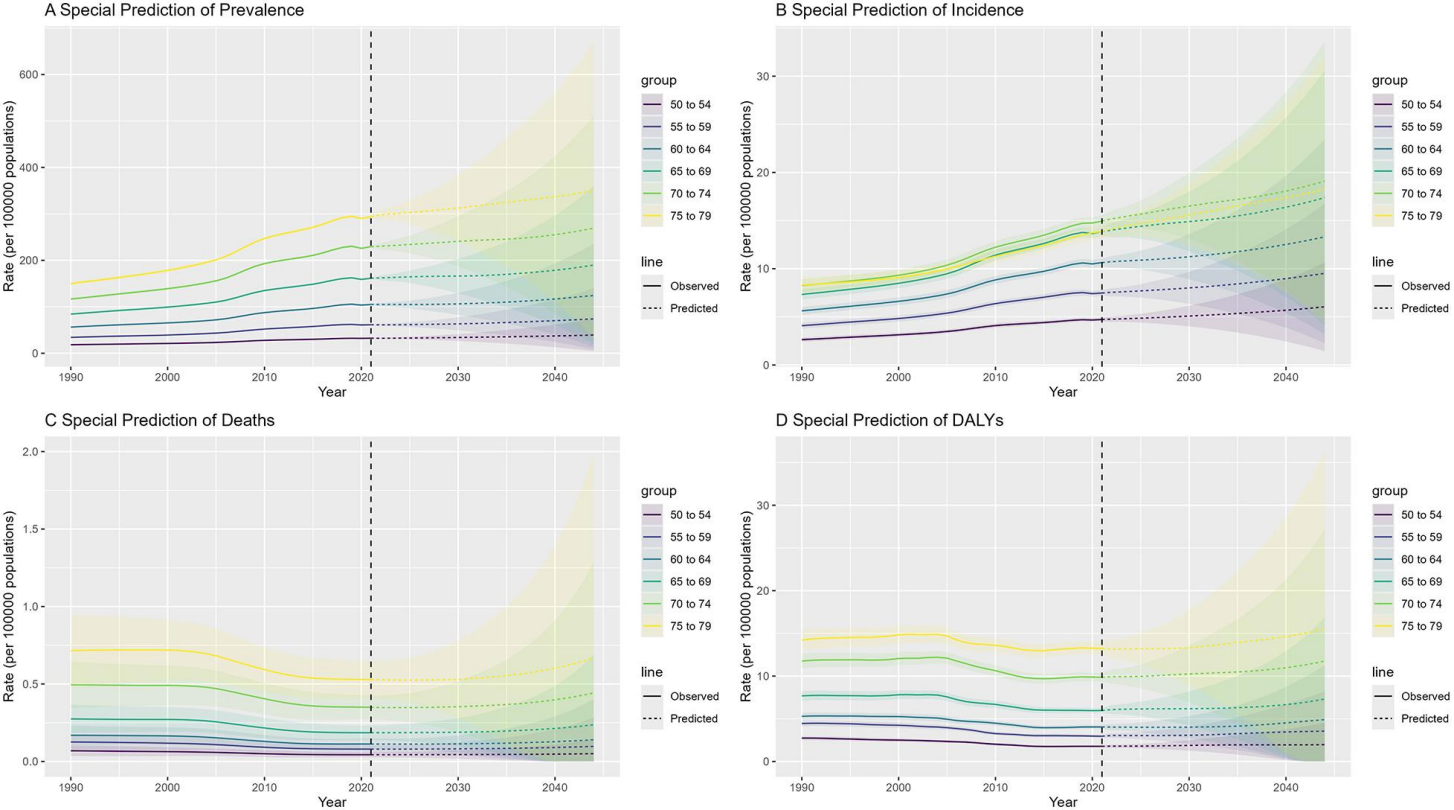
Prediction of NR-CAVD by the BAPC for special age groups in China, including the 50–80-year age group. In the figure, each epidemiological indicator is stratified by age group. The lines indicate the age-standardized rates, and the shaded areas denote the 95% CI. (**A**) prevalence, (**B**) incidence, (**C**) deaths, and (**D**) DALYs.

Within the high-risk 50–80-year subgroup, the 70–74-year age group is predicted to maintain the highest incidence of NR-CAVD, reaching 19 cases per 100,000 by 2050 (95% CI: 4.6–33.6, Figure 6). Interestingly, the mortality curve is expected to decline initially before rising again near mid-century, suggesting that medical improvements may temporarily offset—but not fully eliminate—the long-term impact of population aging.

These projections emphasize that, despite declining fatality, NR-CAVD will remain a growing chronic disease burden in China’s aging population, requiring proactive prevention strategies and adaptation of the healthcare system.

## 4 Discussion

### 4.1 Principal findings

This nationwide analysis of NR-CAVD in China, based on data from the GBD 2021 study, yielded several important findings. First, from 1990 to 2021, both the prevalence and incidence of NR-CAVD increased steadily, while mortality and DALYs declined. Second, the disease burden was predominantly concentrated among adults aged 50 years and older, with men showing higher age-standardized rates than women. Third, APC and Joinpoint analyses identified 2005 as a critical inflection point associated with accelerated disease trends. Finally, Bayesian projections suggest that the NR-CAVD burden will continue to rise through 2050, largely driven by population aging.

Taken together, these findings indicate that NR-CAVD has emerged as a growing non-communicable disease burden in China (17–20). Although mortality has declined with advances in cardiac imaging, interventional valve therapy, and perioperative management, the expanding aging population ensures that the total number of patients will continue to increase in the coming decades.

### 4.2 Comparison with previous studies

Our results align with global analyses demonstrating a consistent upward trajectory in the prevalence of calcific aortic valve disease, particularly in middle- and high-income countries. Data from the GBD 2019 project also identified CAVD as one of the fastest-growing contributors to cardiovascular deaths and DALYs worldwide. However, compared with countries in the high-SDI category, China’s age-standardized incidence and mortality remain at moderate-to-low levels—reflecting both later onset of degenerative valve disease and ongoing differences in healthcare access.

A notable finding of our study is that the NR-CAVD incidence in China increased at a faster rate (EAPC = 2.03%) than the global average (EAPC = 0.88%), suggesting that rapid population aging and improved disease recognition have contributed more strongly in China. These results expand upon previous national analyses that were limited to earlier GBD cycles (1990–2019) and lacked predictive modeling (10).

### 4.3 Possible mechanisms and epidemiological interpretation

The steady rise in NR-CAVD prevalence reflects the complex interplay between biological aging, environmental exposures, and transformations in healthcare systems. Age-related calcification of the aortic valve is a complex process involving chronic inflammation, endothelial dysfunction, and osteogenic differentiation of valvular interstitial cells (3, 21). Emerging evidence also indicates that age-related valvular interstitial cell senescence and calcific deposition are key mechanisms underlying CAVD development. In this process, methionine sulfoxide reductase A (MSRA) has been shown to mitigate valvular senescence by inhibiting the TLR2/NF- κB pathway (22). Another study demonstrated that Schisandrol B promotes the ubiquitination and degradation of p53, thereby suppressing p53-mediated inflammatory and senescence responses during aortic valve calcification (23). Increased life expectancy and the decline in rheumatic valve disease have shifted the national disease spectrum from inflammatory to degenerative etiologies.

The observed gender disparity—characterized by higher incidence and mortality in men—may be attributed to several mechanisms (24–26). Men typically exhibit greater valvular calcification for a given degree of stenosis, potentially due to hormonal and metabolic differences that influence calcium deposition and lipid oxidation within the aortic valve. Imaging studies using multidetector CT have confirmed significantly higher calcification volumes in men than in women at comparable levels of hemodynamic severity (27). Nevertheless, mortality among older women remains high, consistent with global patterns showing greater frailty and a higher burden of comorbidities among aging women.

Previous studies have found that over the past three decades, the steepest increase in the prevalence of NR-CAVD has been reported in East Asia, with China accounting for the largest number of affected individuals. Between 1990 and 2019, population-attributable fractions related to high sodium intake and high lead exposure remained low and relatively stable, with similar trends observed across both sexes and all age groups (28). However, these analyses did not focus on the high-SDI countries within East Asia. Therefore, we plan to extend our investigation to Japan, South Korea, and other similar settings in the near future.

In addition, improvements in echocardiographic screening and diagnostic sensitivity after 2005 likely contributed to the observed inflection in incidence trends. The expanded use of cardiac ultrasound in secondary and tertiary hospitals increased detection of asymptomatic cases, while progress in surgical and transcatheter valve replacement (TAVR) has reduced DALYs by improving long-term survival and functional outcomes (29–31).

### 4.4 Methodological considerations and limitations

Several limitations should be acknowledged when interpreting our findings. First, estimates from the GBD 2021 study rely on model-based syntheses of heterogeneous data sources. Underreporting and misclassification of NR-CAVD, particularly in underdeveloped provinces, may introduce residual uncertainty. Second, our analysis did not include individual-level risk factors such as hypertension, dyslipidemia, or genetic predisposition, all of which are known to influence CAVD progression. Future integrative studies combining population-based surveillance with molecular or imaging data are needed to clarify these associations. Third, although Bayesian age–period–cohort modeling improves forecast stability, projections remain subject to uncertainties from demographic shifts and policy changes. Finally, this study focused solely on national-level estimates; regional variations within China—arising from socioeconomic, lifestyle, and healthcare disparities—should be explored in subsequent work. Despite these limitations, the strengths of this study include its use of the most recent GBD dataset; application of a comprehensive modeling framework combining APC, Joinpoint, and Bayesian approaches; and provision of the first national-level forecast of NR-CAVD burden through 2050.

Apart from the potential shortcomings of the mathematical modeling itself, the inherent limitations of the sample statistics should not be overlooked. First, although we focused exclusively on the epidemiological trends of NR-CAVD in China and referenced the ICD-10 codes used domestically, insufficient data quality and quantity in some regions may have affected the accuracy of our estimates. In addition, because of the country’s vast population base, a considerable number of asymptomatic, potential NR-CAVD cases are likely to have escaped detection, suggesting that the prevalence reported here may underestimate the true disease burden.

### 4.5 Public health and clinical implications

The growing prevalence of NR-CAVD has critical implications for China’s healthcare system. As individuals aged ≥65 years now exceeds 14% of the national population, the burden of degenerative valve disease is expected to expand sharply. Early screening of at-risk adults, optimization of cardiovascular risk management, and expansion of interventional valve therapy will be essential to mitigate disease progression.

From a policy perspective, integrating NR-CAVD into broader non-communicable disease control programs may improve resource allocation and reduce future DALYs. Regional registries and long-term cohort studies should be established to track disease evolution and treatment outcomes. In clinical practice, a multidisciplinary strategy combining cardiology, cardiac surgery, and geriatric care will be vital to address the complex needs of this growing patient population.

### 4.6 Future directions

Future research should focus on three aspects: (1) mechanistic studies to elucidate the cellular and molecular pathways driving valvular calcification in aging populations, (2) risk prediction models that integrate multi-omic biomarkers and imaging phenotypes to enable early detection, and (3) health policy modeling to evaluate the cost-effectiveness of nationwide NR-CAVD screening and prevention programs.

Such efforts will help transform NR-CAVD management from a reactive, treatment-oriented paradigm to a proactive, prevention-centered approach.

## 5 Conclusions

This comprehensive analysis provides the first nationwide overview of NR-CAVD burden in China based on the most recent GBD 2021 data. Over the past three decades, age-standardized prevalence and incidence of NR-CAVD have risen steadily, while mortality and DALYs have gradually declined. The disease predominantly affects older adults—particularly men aged 65 years and above—and exhibits a clear upward trajectory that is projected to continue through 2050.

Our APC and Bayesian modeling results indicate that demographic aging and improved disease recognition are the major drivers of this trend. Although advances in valve intervention and clinical management have reduced mortality, the absolute number of NR-CAVD cases is expected to continue rising as the elderly population expands.

These findings underscore the urgent need for an integrated public health response that includes early screening of high-risk individuals, enhanced cardiovascular risk control, and equitable access to surgical and transcatheter valve therapies. In addition, strengthening national registries and implementing long-term surveillance systems will be essential for monitoring the evolving burden and evaluating the effectiveness of intervention strategies.

Ultimately, addressing NR-CAVD within the broader context of cardiovascular aging represents a critical step toward achieving the goals of “Healthy China 2030” and ensuring sustainable cardiovascular health for an aging society.

## Data Availability

Publicly available datasets were analyzed in this study. This data can be found here: https://ghdx.healthdata.org/about-ghdx/about-data-availability.
