# Supplementary material for: Non-rheumatic calcific aortic valve disease in China: findings from the Global Burden of Disease study 2021
Source: Front Cardiovasc Med. 2026 Jan 16;12:1664291. doi: 10.3389/fcvm.2025.1664291 (PMC12855414; doi:10.3389/fcvm.2025.1664291)
Supplement: Supplementary file 1 [file Datasheet1.pdf]

Supplementary Material

1 SUPPLEMENTARY TABLES AND FIGURES

1.1 Tables

Location	CASE Incidence	ASR Incidence	EAPC Incidence 1990-2021
China	54,642(42,317 to 66,939)	2.52(1.95 to 3.06)	2.03%(1.96% to 2.11%)
Global	1,044,370(906,614 to 1,179,672)	12.03(10.43 to 13.56)	0.79%(0.7% to 0.89%)
East Asia	57,704(44,901 to 70,370)	2.57(2.00 to 3.12)	2.03%(1.97% to 2.1%)
Low SDI	9,847(8,381 to 11,323)	1.89(1.61 to 2.16)	0.55%(0.51% to 0.6%)
Low-middle SDI	46,363(38,524 to 54,556)	3.19(2.68 to 3.73)	1.08%(1.04% to 1.12%)
Middle SDI	116,302(94,895 to 139,288)	4.21(3.43 to 5.01)	1.59%(1.53% to 1.65%)
High-middle SDI	244,577(208,640 to 280,897)	12.13(10.38 to 13.88)	1.2%(1.08% to 1.31%)
High SDI	625,945(542,702 to 701,339)	29.98(26.09 to 33.53)	1.1%(1% to 1.2%)

Table S1. Incidence for 2021 across different locations and SDI categories

Location	CASE Deaths	ASR Deaths	EAPC Deaths 1990-2021
China	1,334(1,030 to 1,786)	0.07(0.06 to 0.10)	-1.34%(-1.56% to -1.12%)
Global	142,205(120,674 to 155,574)	1.83(1.54 to 2.00)	-0.05%(-0.16% to 0.05%)
East Asia	1,757(1,421 to 2,248)	0.10(0.08 to 0.12)	-1.33%(-1.88% to -0.77%)
Low SDI	3,951(2,656 to 5,097)	0.98(0.64 to 1.31)	0.54%(0.37% to 0.7%)
Low-middle SDI	10,222(8,152 to 12,208)	0.84(0.67 to 1.01)	0.78%(0.7% to 0.85%)
Middle SDI	11,639(10,228 to 13,303)	0.49(0.43 to 0.57)	0%(-0.09% to 0.1%)
High-middle SDI	23,645(20,185 to 25,780)	1.27(1.08 to 1.39)	1.01%(0.77% to 1.25%)
High SDI	92,578(74,403 to 102,070)	3.43(2.81 to 3.76)	-0.19%(-0.33% to -0.04%)

Table S2. Deaths for 2021 across different locations and SDI categories

Location	CASE DALYs	ASR DALYs	EAPC DALYs 1990-2021
China	36,638(28,314 to 47,287)	1.92(1.49 to 2.48)	-1.06%(-1.22% to -0.9%)
Global	2,242,999(2,004,168 to 2,459,248)	27.74(24.67 to 30.48)	-0.38%(-0.48% to -0.28%)
East Asia	43,823(35,365 to 55,392)	2.25(1.83 to 2.82)	-1.13%(-1.49% to -0.76%)
Low SDI	101,674(68,877 to 130,980)	19.33(13.08 to 24.89)	0.33%(0.2% to 0.45%)
Low-middle SDI	245,089(199,019 to 292,246)	17.20(13.85 to 20.50)	0.63%(0.58% to 0.67%)
Middle SDI	276,277(242,966 to 316,115)	10.76(9.48 to 12.35)	-0.14%(-0.24% to -0.05%)
High-middle SDI	394,694(352,936 to 434,424)	21.00(18.75 to 23.06)	0.46%(0.29% to 0.62%)
High SDI	1,222,299(1,042,435 to 1,341,835)	50.38(44.16 to 54.83)	-0.65%(-0.82% to -0.48%)

Table S3. DALYs for 2021 across different locations and SDI categories

1.2 Figures

Age	CASE Incidence	ASR Incidence	EAPC Incidence 1990-2021
15 to 19	31(16 to 50)	0.04(0.02 to 0.07)	1.81%(1.71% to 1.92%)
20 to 24	94(49 to 152)	0.13(0.07 to 0.21)	1.87%(1.77% to 1.97%)
25 to 29	188(99 to 302)	0.22(0.12 to 0.35)	1.85%(1.73% to 1.97%)
30 to 34	366(193 to 587)	0.30(0.16 to 0.49)	1.79%(1.65% to 1.93%)
35 to 39	411(216 to 658)	0.39(0.20 to 0.62)	1.75%(1.62% to 1.89%)
40 to 44	1,071(628 to 1,703)	1.17(0.69 to 1.86)	1.83%(1.74% to 1.92%)
45 to 49	2,895(1341 to 5,238)	2.62(1.22 to 4.75)	1.85%(1.76% to 1.94%)
50 to 54	5,707(3,729 to 8,010)	4.72(3.09 to 6.63)	2.01%(1.88% to 2.13%)
55 to 59	8,182(4,743 to 12,760)	7.44(4.31 to 11.61)	2.15%(2% to 2.3%)
60 to 64	7,753(5,205 to 11,265)	10.62(7.13 to 15.43)	2.3%(2.16% to 2.44%)
65 to 69	10,629(6,499 to 15,720)	13.86(8.47 to 20.50)	2.47%(2.34% to 2.6%)
70 to 74	7,937(5,060 to 11,658)	14.89(9.49 to 21.88)	2.34%(2.25% to 2.42%)
75 to 79	4,538(2,661 to 6,742)	13.70(8.04 to 20.36)	1.9%(1.86% to 1.95%)
80 to 84	2,718(1,415 to 4,224)	13.73(7.15 to 21.34)	1.4%(1.28% to 1.52%)
85 to 89	1,414(739 to 2,358)	14.85(7.76 to 24.76)	0.93%(0.75% to 1.11%)
90 to 94	541(274 to 902)	18.47(9.35 to 30.77)	0.6%(0.39% to 0.8%)
95 plus	159(56 to 322)	24.96(8.91 to 50.49)	0.5%(0.3% to 0.71%)

Table S4. Incidence by Age Group of China

Age	CASE Deaths	ASR Deaths	EAPC Deaths 1990-2021
15 to 19	4(2 to 5)	0.01(0.00 to 0.01)	-3.59%(-3.94% to -3.23%)
20 to 24	6(4 to 9)	0.01(0.01 to 0.01)	-2.07%(-2.41% to -1.72%)
25 to 29	9(7 to 11)	0.01(0.01 to 0.01)	-1.36%(-1.6% to -1.11%)
30 to 34	16(12 to 23)	0.01(0.01 to 0.02)	-1.71%(-1.94% to -1.47%)
35 to 39	20(15 to 27)	0.02(0.01 to 0.03)	-1.9%(-2.2% to -1.59%)
40 to 44	22(16 to 29)	0.02(0.02 to 0.03)	-1.8%(-2.09% to -1.52%)
45 to 49	36(25 to 47)	0.03(0.02 to 0.04)	-1.34%(-1.48% to -1.19%)
50 to 54	52(37 to 72)	0.04(0.03 to 0.06)	-1.95%(-2.13% to -1.77%)
55 to 59	85(62 to 120)	0.08(0.06 to 0.11)	-2.06%(-2.24% to -1.87%)
60 to 64	85(63 to 118)	0.12(0.09 to 0.16)	-1.49%(-1.62% to -1.35%)
65 to 69	140(105 to 190)	0.18(0.14 to 0.25)	-1.75%(-1.95% to -1.55%)
70 to 74	187(142 to 255)	0.35(0.27 to 0.48)	-1.57%(-1.79% to -1.35%)
75 to 79	180(136 to 247)	0.55(0.41 to 0.75)	-1.1%(-1.25% to -0.96%)
80 to 84	181(137 to 249)	0.92(0.69 to 1.26)	-0.8%(-0.98% to -0.62%)
85 to 89	182(141 to 240)	1.92(1.49 to 2.53)	-0.73%(-1.08% to -0.38%)
90 to 94	96(69 to 138)	3.28(2.39 to 4.72)	-1.35%(-1.69% to -1.01%)
95 plus	26(16 to 40)	4.17(2.63 to 6.26)	-1.54%(-1.9% to -1.19%)

Table S5. Deaths by Age Group of China

Age	CASE DALYs	ASR DALYs	EAPC DALYs 1990-2021
15 to 19	291(211 to 410)	0.39(0.28 to 0.55)	-3.6%(-3.95% to -3.24%)
20 to 24	440(324 to 640)	0.60(0.44 to 0.88)	-2.07%(-2.42% to -1.72%)
25 to 29	577(446 to 748)	0.67(0.52 to 0.87)	-1.36%(-1.6% to -1.12%)
30 to 34	979(742 to 1,369)	0.81(0.61 to 1.13)	-1.7%(-1.93% to -1.47%)
35 to 39	1,095(839 to 1,463)	1.03(0.79 to 1.38)	-1.89%(-2.19% to -1.58%)
40 to 44	1,086(792 to 1,430)	1.19(0.87 to 1.56)	-1.79%(-2.08% to -1.51%)
45 to 49	1,593(1,150 to 2,120)	1.44(1.04 to 1.92)	-1.3%(-1.44% to -1.16%)
50 to 54	2,137(1,596 to 2,890)	1.77(1.32 to 2.39)	-1.81%(-1.97% to -1.64%)
55 to 59	3,188(2,353 to 4,381)	2.90(2.14 to 3.98)	-1.82%(-1.99% to -1.64%)
60 to 64	2,983(2,251 to 3,929)	4.09(3.08 to 5.38)	-1.12%(-1.22% to -1.01%)
65 to 69	4,568(3,445 to 6,121)	5.96(4.49 to 7.98)	-1.14%(-1.28% to -0.99%)
70 to 74	5,261(3,950 to 6,990)	9.87(7.41 to 13.12)	-0.88%(-1.03% to -0.73%)
75 to 79	4,401(3,285 to 5,892)	13.29(9.92 to 17.79)	-0.36%(-0.44% to -0.27%)
80 to 84	3,646(2,760 to 4,918)	18.43(13.95 to 24.85)	-0.04%(-0.15% to 0.07%)
85 to 89	2,760(2,101 to 3,619)	28.98(22.06 to 38.00)	-0.09%(-0.34% to 0.16%)
90 to 94	1,237(927 to 1,663)	42.22(31.63 to 56.74)	-0.66%(-0.92% to -0.41%)
95 plus	387(278 to 528)	60.57(43.57 to 82.63)	-0.57%(-0.82% to -0.32%)

Table S6. Deaths by Age Group of China

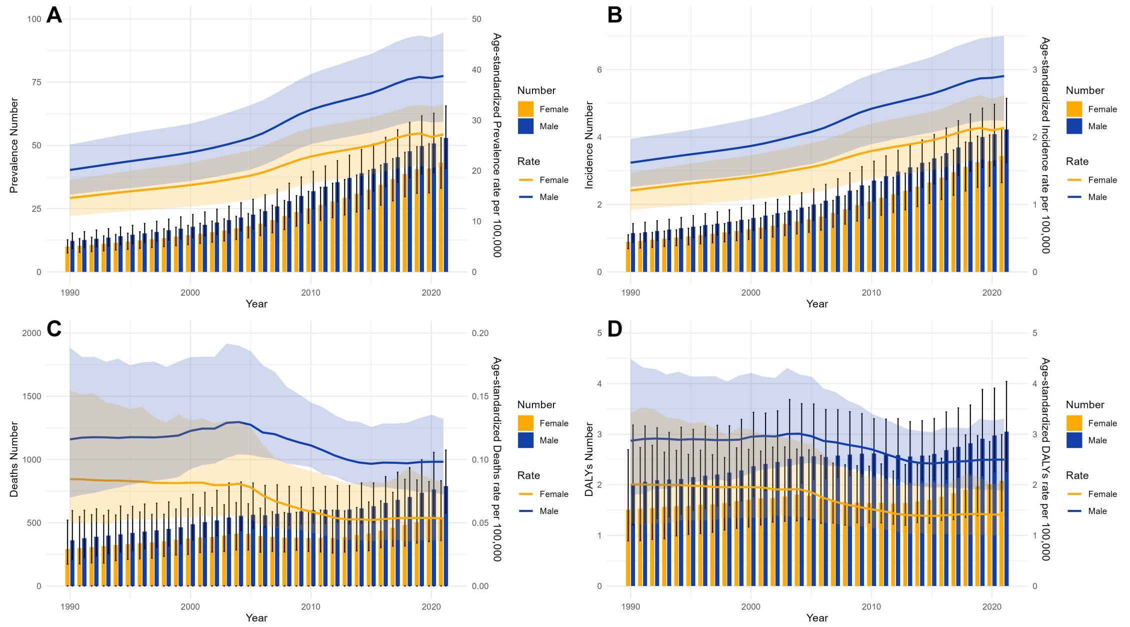

Figure S1. The ASR epidemiological parameters of NR-CAVD for age groups in China, compared by gender in 1990-2021. Bars: number of cases in each age group. Error Bars: 95% CI of the case number. Line: ASR parameters. Shaded Area: 95% CI for the ASR. A: prevalence B: incidence C: deaths D: DALYs

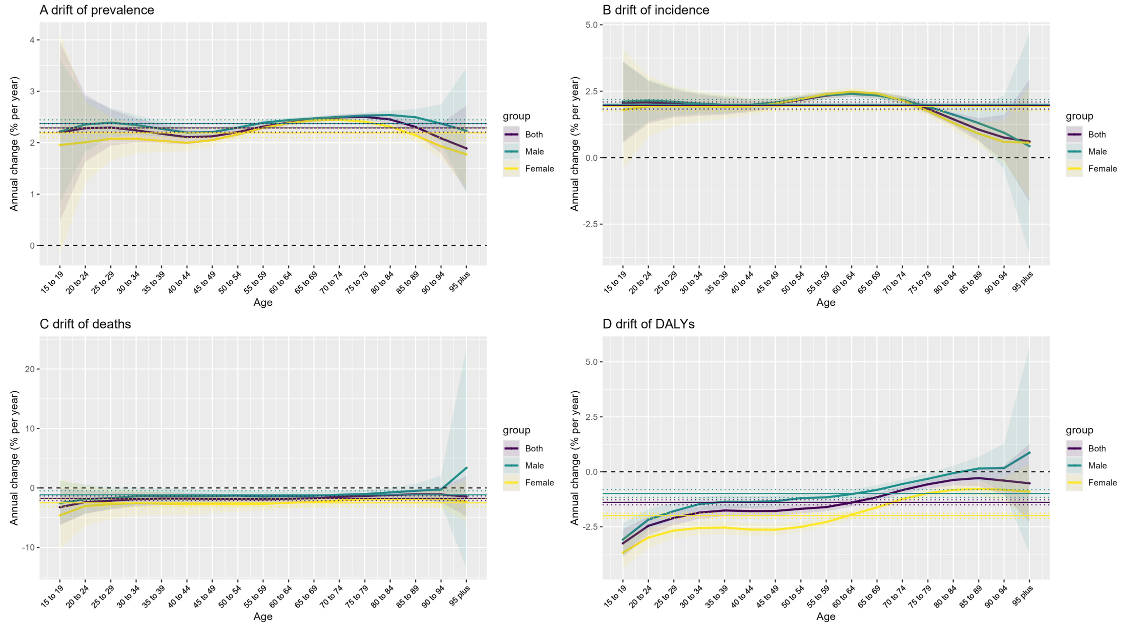

Figure S2. The drift of APC, compared by gender in 2021. A: prevalence B: incidence C: deaths D: DALYs

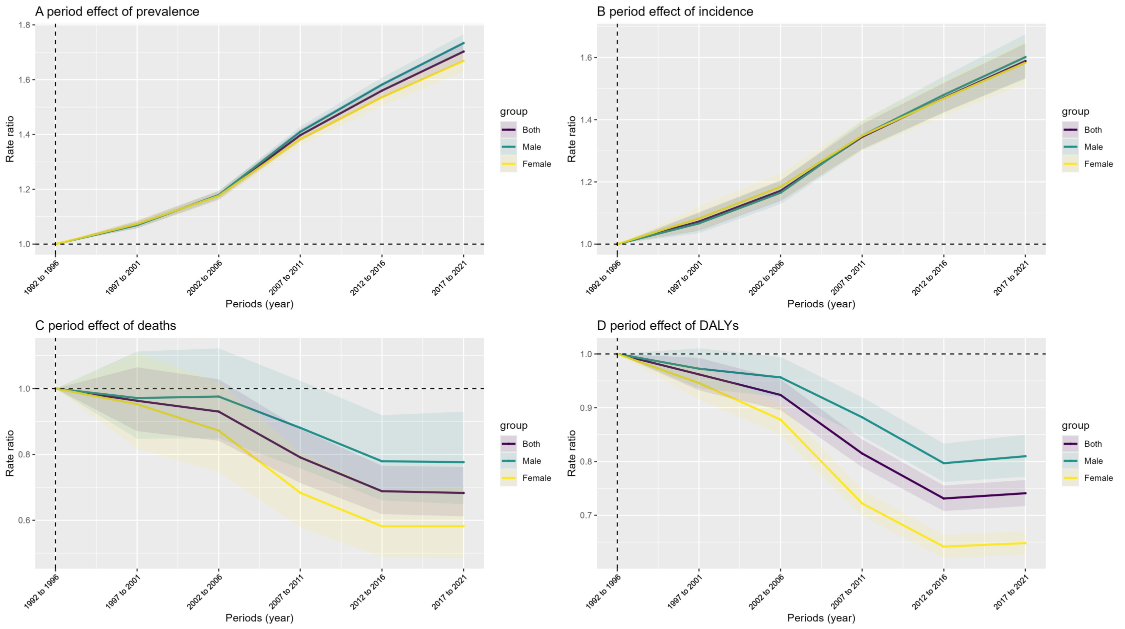

Figure S3. The period effect of APC, compared by gender in 2021. A: prevalence B: incidence C: deaths D: DALYs

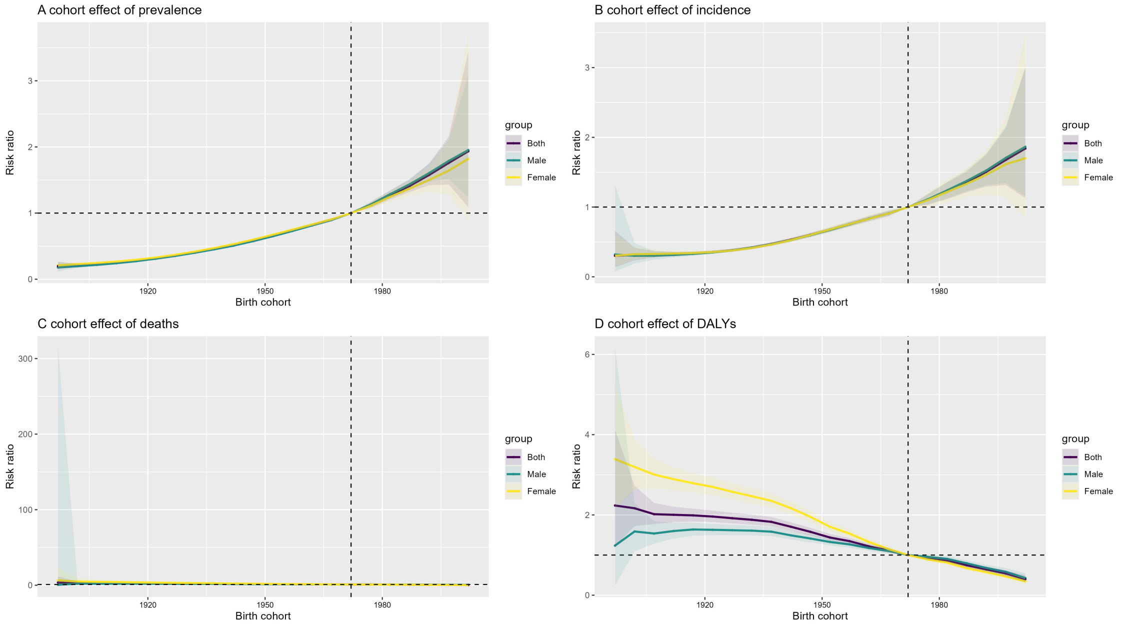

Figure S4. The cohort effect of APC, compared by gender in 2021. A: prevalence B: incidence C: deaths D: DALYs

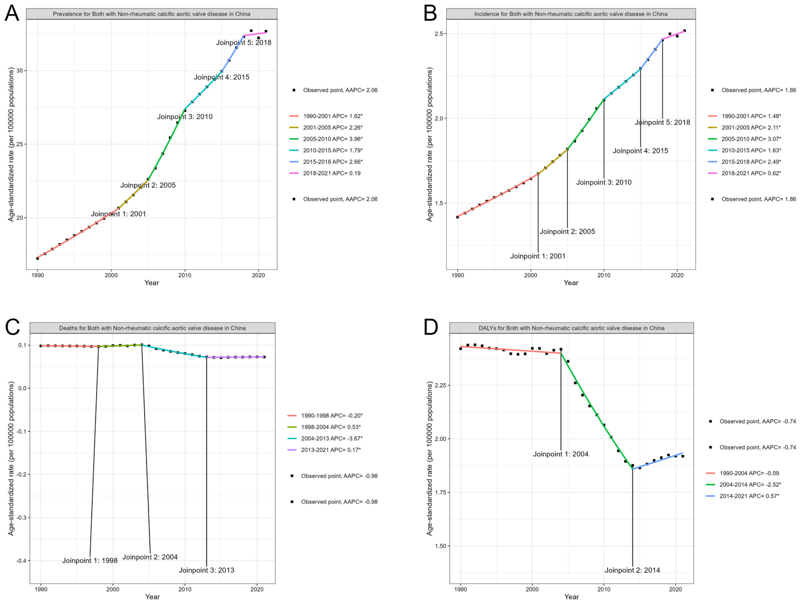

Figure S5. The Joinpoint predication of NR-CAVD in China. The determination of the number of change points adopts the Monte Carlo permutation test. We set the maximum potential number of change points to 5 and the minimum to 0. The permutation test starts with the number of change points $k = 0$ and $k_{\max} = 5$. If $k \neq k_{\max}$, then set $k = k + 1$ and continue the test until the model corresponding to $k = k_{\max}$ is selected as the optimal model. A: prevalence B: incidence C: deaths D: DALYs
